# The Epidemiological Profile and Predisposing Factors of Microbial Keratitis Over a 10-Year Period at a Tertiary Hospital in Central Saudi Arabia

**DOI:** 10.7759/cureus.45433

**Published:** 2023-09-18

**Authors:** Abdulaziz M Al Hadlaq, Meshari M Al Harbi

**Affiliations:** 1 Ophthalmology, College of Medicine, Qassim University, Buraydah, SAU; 2 Ophthalmology, King Fahad Specialist Hospital, Buraydah, Buraydah, SAU

**Keywords:** ocular trauma, hypopyon, contact lens, corneal ulcer, microbial keratitis

## Abstract

Purpose: To evaluate the demographic and predisposing factors and clinical presentation of microbial keratitis (MK) patients over 10 years at a tertiary hospital in central Saudi Arabia.

Method: In 2020, a retrospective review of data from clinical and pathology departments from 2010 to 2019 was conducted. Demography includes age, gender, residence, and the risk factor of microbial keratitis, clinical features, and organism profile as number and percentage.

Result: We reviewed 181 eyes of 179 patients with microbial keratitis. The mean age was 40.1 years. Contact lens usage (55; 30%), ocular trauma (30; 16.5%), and ocular surface diseases (42; 23.2%) were the main predisposing factors. Hypopyon was noted in 60 (33%) eyes, impaired vision at presentation was observed in 78 (43%) eyes, and endophthalmitis with microbial keratitis was noted in eight (4.4%) eyes at presentation. Ninety-eight (54,1%) cases were culture positive, while gram-positive organisms were seen in 50 (27.6%) eyes, gram-negative organisms in 35 (19.3%) patients, and fungi in 13 (7.2%) patients. Microbial keratitis was central in 67 (37%), paracentral in 94 (52%), and peripheral in 20 (11.3%) patients. All instances of fungal keratitis occurred in the eyes of men who work in the agriculture field.

Conclusion: Standard operating procedures to manage microbial keratitis at primary and secondary eye care levels are recommended. Health promotion to prevent trauma, appropriate use of contact lens, and early treatment could prevent the incidence of microbial keratitis.

## Introduction

Microbial keratitis (MK) is a potentially vision-threatening condition, with approximately 2 million people annually affected worldwide. It is mainly caused by bacteria in developed countries and fungi in developing countries [1]. In large countries like the USA, causative agents of microbial keratitis vary depending on region, with fungal keratitis being more prevalent in the southern states [2].

Wide use of different types of contact lenses in recent decades, both for clear vision and for cosmetic appearance, has increased the risk of microbial keratitis. As a consequence, it is vital to understand the condition’s risk factors, presentation, and organism profile. Other risk factors of microbial keratitis include ocular trauma and the presence of ocular surface disease [3-5]. A number of studies with a high level of evidence have been undertaken on this issue, documenting geographic variation in organism profiles and causative agents [6,7].

In Middle Eastern countries, the past literature shows the microbial keratitis organism profile in the United Arab Emirates, and the most common organism was *Pseudomonas aeruginosa* [8]. Publications on microbial keratitis in Saudi Arabia have included the use of soft contact lenses in children, and in a study at a tertiary hospital in the center of the country, the most common predisposing factor was ocular trauma (39.7%) [9-24]. In this paper, the study aimed to contribute to the existing research by presenting the microbial keratitis profile, causative agents, and underlying risk factors reported over a 10-year period at Security Forces Hospital, Riyadh, Saudi Arabia. This institution has a well-equipped and qualified ophthalmology department and pathology unit.

## Materials and methods

Patients presenting to emergency or routine clinics with a provisional diagnosis of microbial keratitis during the study period were included. The demographic information sourced included age, gender, and eye involved. A detailed history was obtained to understand predisposing factors like the use of contact lenses, dry eye, previous ocular surgery, ocular trauma, and lid abnormalities (e.g., blepharitis, trachoma, trichiasis, and incomplete lid closure). The history of previous use of topical medications (e.g., antibiotics, steroids, cycloplegics, and traditional medicine) was also obtained.

The presenting symptoms and duration were obtained from records on clinical examination, which included slit-lamp bio-microscopy (Topcon, USA), presented visual acuity notations using Snellen’s visual acuity chart, perception of hand movement, and perception of light from all angles. The location, extent, and depth of associated infiltrate of corneal epithelial erosion and corneal ulcer were noted without and then with corneal staining. The presence of aqueous flare, iris inflammation status of a pupil, and keratic precipitates (KPs) on the posterior surface of the cornea were examined.

The data were collected on a pretested data collection form. They were then transferred into a Statistical Package for Social Sciences (SPSS 25) spreadsheet (IMB, NY, USA). The qualitative variables were presented as numbers and percentages, while the quantitative variable was plotted to study its normality. If the distribution was normal, we presented it as mean and standard deviation, whereas for the non-normal distribution of variables, we calculated the median and interquartile range.

An approval from the institutional review board was taken under accreditation number: H-01-R-069), the medical records of each patient admitted at Security Forces Hospital, Riyadh, Saudi Arabia, for the treatment of microbial keratitis from 2010 until the end of 2019 were retrospectively reviewed. The tenets of the Helsinki Declaration were strictly adhered to during each stage of the research.

## Results

The results included 181 eyes of 179 patients with a provisional diagnosis of microbial keratitis. Their mean age was 40.1 ± 20.7 years (range: 1 to 100), and there were 95 males (52.7%) and 86 (47.3%) females. The information on predisposing factors resulting in microbial keratitis in the 181 eyes is given in Table 1. Contact lens usage was possibly a responsible factor in as many as one-third of the eyes. Ocular trauma resulted in microbial keratitis in one of six eyes. As many as 102 patients were suffering from diabetes, and 75% of them had poor glycemic control (hemoglobin A1C >10).

**Table 1:** Profile of predisposing factors among clinically suspected microbial keratitis cases

					Number		Percentage	
Contact lens				Extended wear	47		26.0	
				Daily wear	7		3.9	
				Hard type	1		0.66	
Ocular trauma						30		16.5
		Keratoplasty				15		8.3
		Cataract				8		4.4
Previous ocular surgery		Glaucoma				3		1.7
		Vitreoretinal surgery				11		6.1
		Refractive surgery				2		1.1
Ocular surface disorder	Blepharitis					19	10.5	
	Trachoma					7	3.1	
	Trichiasis					2	1.1	
	Incomplete lid closure					6	3.3	
	Dryness					8	4.4	

The mean time interval of symptom development and presentation at our institution was 7.9 ± 3.6 days (range: 1 to 60 days). In 45 [24.8%] patients, topical treatment was already initiated by the family physician or referring ophthalmologist. Pain and redness were present in around 172 (95%) eyes, but visual complaints were present in only 78 (43%) eyes. Epithelial erosion was present in all cases, and corneal edema was present in 175 (96.7%). The corneal ulcer was central in 67 (37%), paracentral in 94 (52%), and peripheral in 20 (11.3%) eyes. The average size of the infiltrate was 7.0 mm (range: 0.3 mm to 11.0 mm). Hypopyon was present in 60 (33%) eyes.

The microbial profile is given in Table 2. A total of 83 (45.9%) eyes had no organism isolated from the specimen sent for culture and sensitivity. More than half of the eyes that had a positive report were found to have gram-positive bacteria as the causative agent. Fungus was isolated in 13 (13.2%) eyes with microbial keratitis confirmed by laboratory tests.

**Table 2:** Microbial profile of eyes with clinically suspected corneal ulcer

Microorganism	Number	Percentage
Culture positive	98	54.1
Culture negative	83	45.9
Bacterial isolate	85	86.7
Gram positive	50	51
Staphylococcus aureus	25	50
Streptococcus pneumoniae	21	42
Corynebacterium species	1	0.2
Others	3	
Gram negative	35	35.7
Pseudomonas aeruginosa	28	80
*Serratia* spp.	3	8.5
Moraxella species	2	5.7
Klebsiella species	2	5.7
Fungus	13	13.2
Fusarium species	6	46.1
Aspergillus species	4	23
Cryptococcus	1	7
Candida	2	15.3

The relationship between predisposing factors and type of organisms found in the ulcers is shown in Table 3. There was a significant association between organisms and the predisposing factor (c2 = 35, degree of freedom = 12, P = 0.0005).

**Table 3:** Predisposing factor and microbial of corneal ulcer

Validation	No growth	Multiple	Fungus	Bacteria	Factor
c2 = 35, degree of freedom = 12, P = 0.0005	18	2	5	2	Trauma
	23	4	0	31	Contact lens
	8	0	2	8	Ocular surface disorder
	8	3	0	16	Previous ocular surgeries
	26	2	6	14	Unknown

The profile of microbial keratitis-causing organisms in the present study is compared with that mentioned in the literature (Table 4).

**Table 4:** Organism profile of corneal ulcer published in the literature and present study

Predisposing	Culture	Mean	Year	N	Country	Study
Prior ocular surgery	85	50	2009	103	Kingdom of Saudi Arabia	Al-Shehri et al. [12]
Prior contact lens	39	51	2009	285	Bahrain	Al-Yousuf [13]
Trauma (57%)	33	-	1993	200	Kingdom of Saudi Arabia	Al-Mansouri [22]
Contact lens	77.5	40	2011	111	French	Darugar et al. [23]
Trauma (90%)	59	40	2012	150	India	Lin et al. [24]
Trauma (75%)	55		2010	200	India	Kumar et al. [25]
Contact lens	54.5	50	2019	181	Kingdom of Saudi Arabia	Present study

## Discussion

This research paper is an internal audit by cornea specialists treating patients with corneal ulcers. It reflected trends of MK and geographic variation in etiology as well as organisms affecting the urban population of the present study. An increase in the incidence of corneal ulcer cases stresses the urgent need to highlight the importance of primary eye care and health promotion to the general population, especially those suffering from diabetes and other immune-compromised ailments. The rising trend of using contact lenses among the younger generation also needs further investigation in order to study the underlying causes and effects.

Fungal keratitis was not common in our study, but incomplete treatment by blanket antibiotic therapy resulted in no growth detected in the laboratory test. Hence, empirical but newer and less widely used antibiotics were found to be more effective. The epidemiological outcomes of MK noted in the present study were compatible with tertiary eye hospital outcomes in the Saudi capital Riyadh, as well as in Bahrain and Muscat, Oman [12-14]. This reflects similar health-related behavior, as the ocular hygiene of urban populations of these three Gulf countries is responsible for MK. A common strategy to address this issue among GCC countries can therefore be prepared.

Overnight wearing of contact lenses is the main risk for infective keratitis. Emerging trends suggest that MK is increasingly caused by gram-negative organisms, fungi, and acanthamoeba. Resistance in gram-positive organisms to conventional antibiotics in MK has been noted [5]. More than 50% of users have bad hygiene practices when it comes to lens-case cleaning [15]. To combat this problem, research is ongoing to alter lenses and accessories to make them organism resistant [16]. Extended wearing of contact lenses was found to be the main culprit of MK in our study, an observation also noted by Seal et al. [17].

Apart from blepharitis, the results of the current study showed a few ocular surface disorders among patients with MK. Narayanan et al. [18] reported the absence of an association between dry eyes and MK. Apart from trachomatous trichiasis [19], other eyelid infective conditions may be present in eyes with MK, which reflects the poor hygienic practices of such patients, yet their causal association is difficult to establish. In our cohort, ocular trauma, be it accidental or surgically induced, was observed in more than one-third of MK cases. This was also noted in a study in Australia [20]. The intact corneal epithelium seems to act as a protective barrier against invading organisms, but when this barrier is broken by ocular trauma, MK can occur.

The distribution of MK in men and women in the present study was not different. In contrast, men working outdoors and involved in agricultural activities in developing countries are at higher risk of MK, as noted in India [21]. In the USA, more women use contact lenses than males. In the wealthy urban areas of Saudi Arabia, this higher use of contact lenses among women could explain similar risks of MK in the present study [22].

In our study, no organisms were detected in 46% of MK cases. This could be due to the use of antibiotics before patient presentation at our institute. The inability to detect organisms has been noted in both developing and industrialized countries [14,17,21-26]. In the former, this could be due to a lack of resources, while in the latter, it could be due to previous antibiotic treatments. In the gram-positive bacteria group, *Staphylococcus aureus* and *Streptococcus pneumoniae* were the leading organisms, while *Pseudomonas aeruginosa* was the main gram-negative organism in our cohort. The latter were also the primary gram-negative organisms in a study on MK in England [27]. The gram-positive organisms were found to be common results of MK cases in developing countries [6].

In our cohort, there were few instances of fungal keratitis, and the ones that we did discover were linked with a history of ocular trauma (wooden sticks) and diabetes. Surprisingly and reassuringly, there was no case of MK by acanthamoeba in our study despite the wide use of contact lenses by these patients. There were a few limitations to our study. A molecular study is recommended for fast and accurate diagnosis of organisms and species [1]. The lack of availability of such facilities at our hospital cannot be explained, and therefore, further studies are recommended.

Our study was conducted in an urban area that offers free eye care services, and it targeted a population with wide contact lens usage. Thus, information on the profile and causative factors of MK adds epidemiological information to the literature. Health promotion policies to prevent MK, especially among contact lens users, and laying down standard operating procedures to manage MK at primary and secondary eye care facilities in Saudi Arabia, are recommended.

## Conclusions

Overnight use of contact lens was the most significant factor to cause microbial keratitis in our study, followed by ocular trauma. Health promotion policies to prevent microbial keratitis, especially among contact lens users, and laying down standard operating procedures to manage microbial keratitis at primary and secondary eye care facilities in Saudi Arabia, are recommended. By addressing the vision-threatening effects of the use of contact lens wearing and avoidance of eye trauma, microbial keratitis prevalence can be reduced in Saudi Arabia.
